# Supplementary material for: Maternal Stress, Early Life Factors and Infant Salivary Cortisol Levels
Source: Children (Basel). 2022 Apr 27;9(5):623. doi: 10.3390/children9050623 (PMC9139396; doi:10.3390/children9050623)
Supplement: Supplementary file 1 [file children-09-00623-s001.zip › children-1638626-supplementary.pdf]

Article

Maternal Stress, Early Life Factors and Infant Salivary Cortisol Levels

Caroline-Aleksi Olsson Mägi [1,2,*], Åshild Wik Despriee [3,4], Milada Cvancarova Småstuen [4], Catarina Almqvist [2,5], Fuad Bahram [6], Egil Bakkeheim [7], Anders Bjerg [1,8], Kari Glavin [4], Berit Granum [9], Guttorm Haugen [3,10], Gunilla Hedlin [1,2], Christine Monceyron Jonassen [11,12], Karin C. Lødrup Carlsen [3,7], Eva Maria Rehbinder [13], Leif-Bjarte Rolfsjord [7,14], Anne Cathrine Staff [3,10], Håvard Ove Skjerven [3,7], Riyas Vettukattil [3,7], Björn Nordlund [1,2] and Cilla Söderhäll [1,2]

1 Department of Women's and Children's Health, Karolinska Institutet, SE-171 77 Stockholm, Sweden; bjerg.anders@gmail.com (A.B.); gunilla.hedlin@ki.se (G.H.); bjorn.nordlund@ki.se (B.N.); cilla.soderhall@ki.se (C.S.)
2 Astrid Lindgren Children's Hospital, Karolinska University Hospital, SE-171 64 Stockholm, Sweden; catarina.almqvist@ki.se
3 Faculty of Medicine, Institute of Clinical Medicine, University of Oslo, NO-0424 Oslo, Norway; ashild.despriee@vid.no (Å.W.D.); g.n.haugen@medisin.uio.no (G.H.); k.c.l.carlsen@medisin.uio.no (K.C.L.C.); uxnnaf@ous-hf.no (A.C.S.); h.o.skjerven@medisin.uio.no (H.O.S.); riyas.vettukattil@medisin.uio.no (R.V.)
4 Faculty of Health, VID Specialized University, NO-0424 Oslo, Norway; milasm@oslomet.no (M.C.S.); kari.glavin@vid.no (K.G.)
5 Department of Medical Epidemiology and Biostatistics, Karolinska Institutet, SE-171 77 Stockholm, Sweden
6 Research Centre, Stockholm South General Hospital, SE-118 83 Stockholm, Sweden; fuad.bahram@ki.se
7 Division of Paediatric and Adolescent Medicine, Oslo University Hospital, NO-0424 Oslo, Norway; uxbake@ous-hf.no (E.B.); leif.bjarte.rolfsjord@sykehuset-innlandet.no (L.-B.R.)
8 Martina Children's Hospital, SE-114 86 Stockholm, Sweden
9 Department of Chemical Toxicology, Norwegian Institute of Public Health, NO-0213 Oslo, Norway; berit.granum@fhi.no
10 Division of Obstetrics and Gynaecology, Oslo University Hospital, NO-0424 Oslo, Norway
11 Faculty of Chemistry, Biotechnology and Food Science, Norwegian University of Life Sciences, NO-1430 Ås, Norway; christine.monceyron.jonassen@so-hf.no
12 Genetic Unit, Centre for Laboratory Medicine, Østfold Hospital Trust, NO 1714 Kalnes, Norway
13 Department of Dermatology and Vaenerology, Oslo University Hospital, NO-0424 Oslo, Norway; e.m.rehbinder@ous-research.no
14 Department of Paediatric and Adolescent Medicine Elverum, Innlandet Hospital Trust, NO-2381 Brumunddal, Norway
* Correspondence: caroline-aleksi.olsson.magi@ki.se

Citation: Olsson Mägi, C.-A.; Wik Despriee, Å.; Småstuen, M.C.; Almqvist, C.; Bahram, F.; Bakkeheim, E.; Bjerg, A.; Glavin, K.; Granum, B.; Haugen, G.; et al. Maternal Stress, Early Life Factors and Infant Salivary Cortisol Levels. *Children* **2022**, *9*, 623. https://doi.org/10.3390/children9050623

Academic Editor: Evalotte Mörelius

Received: 28 February 2022
Accepted: 20 April 2022
Published: 27 April 2022

Publisher's Note: MDPI stays neutral with regard to jurisdictional claims in published maps and institutional affiliations.

Copyright: © 2022 by the authors. Submitted for possible open access publication under the terms and conditions of the Creative Commons Attribution (CC BY) license (https://creativecommons.org/licenses/by/4.0/).

Supplementary Figure S1.

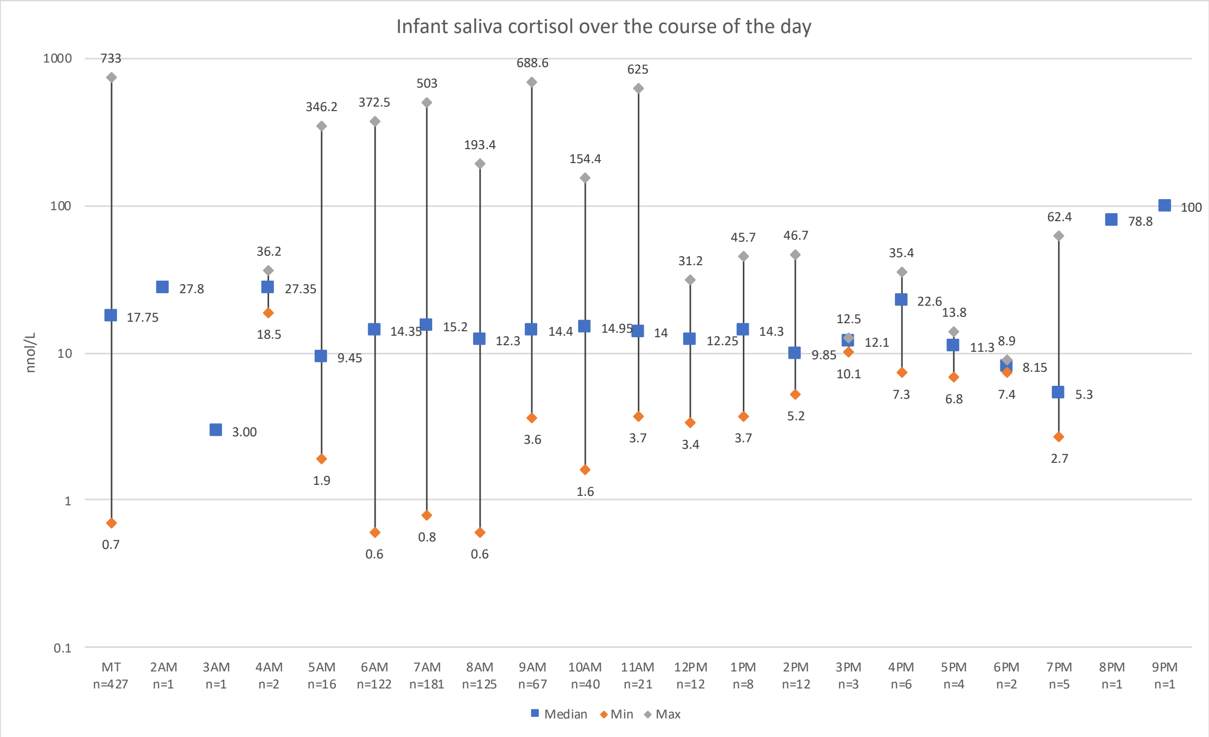

Supplementary Figure S1. Infant saliva cortisol (nmol/L) median levels related to sampling time (*n* = 1057) over 24 h. Infant saliva cortisol values are given in median (blue large squares) and min-max (grey and orange small squares). MT=missing time of saliva sampling (*n* = 427).

Supplementary Table S1. Univariate multinomial logistic regression model analysis of possible associations of factors in pregnancy, birth and early life and infant salivary cortisol at three months of age (n=1057). Results are presented as Odds Ratio (OR), 95 % Confidence intervals (95% CI) and p-value (p). Univariate multinomial logistic reference category <5.50 nmol/L, *n* = 266)

	iSC 5.51-15.35 nmol/L (*n* = 263)			iSC 15.36-24.93 nmol/L (*n* = 265)			iSC >24.94 nmol/L (*n* = 263)		
	OR	95% CI	*p*	OR	95% CI	*p*	OR	95% CI	*p*
Gestational age, birth	1.00	0.99-1.03	0.38	1.00	0.99-1.03	0.48	1.00	0.99-1.03	0.54
Cortisol, sampling age	1.02	0.95-1.09	0.59	1.03	0.96-1.10	0.39	1.04	0.98-1.11	0.20
**Birth weight***	**1.01**	**1.00-1.09**	**0.05**	**1.03**	**1.00-1.07**	**0.09**	**1.05**	**1.01**	**0.02**
Maternal age	0.99	0.95-1.04	0.73	0.98	0.94-1.02	0.34	0.98	0.94-1.02	0.33
Girl	0.98	0.70-1.37	0.9	0.96	0.69-1.35	0.38	0.91	0.64-1.27	0.57
Boy (ref.)	1.00			1.00			1.00		
Induced birth start	1.04	0.69-1.55	0.86	1.07	0.72-1.60	0.74	0.84	0.55-1.27	0.41
Natural birth start (ref.)	1.00			1.00			1.00		
Cesarean section	**0.61**	**0.37-0.99**	**0.05**	0.86	0.54-1.35	0.50	1.20	0.78-1.85	0.41
Vaginal birth (ref.)	1.00			1.00			1.00		
Food intervention	1.16	0.68-2.00	0.58	1.34	0.79-2.28	0.28	1.08	0.63-1.86	0.78
Food and bath intervention	0.84	0.48-1.47	0.55	1.04	0.61-1.78	0.89	1.17	0.69-2.00	0.55
Bath Intervention	1.00	0.59-1.69	1.00	0.77	0.45-1.32	0.34	0.74	0.43-1.27	0.27
No intervention (ref.)	1.00			1.00			1.00		
Not living with a partner	0.54	0.29-1.01	0.05	**0.51**	**0.27-0.95**	**0.03**	**0.48**	**0.25-0.91**	**0.02**
Married/cohabitant (ref.)	1.00			1.00			1.00		

Low family income	0.76	0.48-1.25	0.30	1.01	0.63-1.59	0.99	0.91	0.57-1.44	0.91
Income >600.000 (ref.)	1.00			1.00			1.00		
Low maternal education	0.92	0.51-1.67	0.79	1.28	0.73-2.23	0.39	1.15	0.65-2.03	0.63
University < (ref.)	1.00			1.00			1.00		
First child	1.15	0.80-1.55	0.45	1.07	0.74-1.53	0.73	1.02	0.71-1.46	0.97
Previous children	1.00			1.00			1.00		
Maternal nicotine 3m	0.55	0.24-1.28	0.17	0.91	0.43-1.90	0.80	0.93	0.44-1.94	0.84
No nicotine (ref.)	1.00			1.00			1.00		
Mixed fed	1.03	0.72-1.49	0.86	0.77	0.53-1.11	0.16	**0.66**	**0.45-9.95**	**0.02**
Breastfed only (ref.)	1.00			1.00			1.00		
Colic 3 m	0.90	0.34-2.37	0.83	0.69	0.24-1.96	0.48	0.94	0.36-2.49	0.90
No reported colic (ref.)	1.00			1.00			1.00		
Abdominal pain 3 m	1.04	0.67-1.61	0.86	1.08	0.70-1.68	0.72	1.16	0.75-1.80	0.50
No abdominal pain (ref.)	1.00			1.00			1.00		
PSS 18w	1.02	0.99-1.04	0.19	1.00	0.98-1.02	0.96	1.00	0.98-1.03	0.93
PSS 34w	1.01	0.99-1.04	0.33	1.00	0.98-1.03	0.74	1.00	0.98-1.02	0.86
PSS 3m	1.00	0.98-1.03	0.85	0.99	0.97-1.02	0.61	0.99	0.96-1.01	0.35
High PSS 18w 29	1.10	0.68-1.77	0.71	1.07	0.66-1.73	0.79	0.93	0.57-1.53	0.79
**High PSS 34w** 29	1.07	0.63-1.84	0.80	**1.57**	**0.95-2.61**	**0.08**	0.98	0.57-1.70	0.95
High PSS 3m 29	1.19	0.63-2.26	0.60	0.87	0.44-1.74	0.69	0.79	0.38-1.57	0.47
Low stress <29 (ref.)	1.00			1.00			1.00		

Supplementary Table S2. Mann–Whitney U-test (pairwise with reference categorie (ref)) of background factors in pregnancy, at birth and by three months infant age and differences in median infant saliva cortisol at three moths age. Number are presented as number (*n*), present (%) and *p*-value. Median saliva cortisol and inter quartile range (IQR). (n = 1057).

		Study cohort (n = 1057)		*p*-value
		n (%)	median (IQR)	
Infant sex	Boy (ref)	543 (51.4)	15.50 (15.90)	
	Girl	514 (48.6)	15.10 (14.58)	0.69
Birth start	Spontaneous (ref)	803 (75.9)	15.50 (16.87)	
	Medical induced	240 (22.7)	14.90 (13.42)	0.59
Birth method	Vaginal (ref)	881 (83.3)	15.50 (16.87)	
	Caesarean section	176(16.7)	14.90 (13.42)	0.16
PreventADALL intervention group	No intervention (ref)	214 (20.5)	16.00 (16.60)	
	Bath	212 (20.1)	13.60 (13.70)	0.25
	Food	227 (21.5)	16.50 (18.30)	0.32
	Bath/Food	209 (19.8)	16.50 (14.90)	0.47
Marital status	Married/cohabitant (ref)	979 (92.6)	15.50 (15.40)	
	Not living with a partner	78 (7.4)	12.10 (14.10)	0.08
Yearly family income	>600.000 (ref)	894 (84.6)	15.20 (15.33)	
	Less than 600.000	163 (15.4)	15.85 (16.33)	0.72
Maternal education	University or higher (ref)	950 (89.9)	15.2 (15.00)	
	High school	107 (10.1)	17.50 (16.83)	0.30
Parity	First child (ref)	341 (32.3)	15.60 (17.00)	
	One or more children	716 (67.7)	15.30 (14.75)	0.88

Maternal nicotine use by 3-month	No (ref)	880 (83.3)	14.8 (14.66)	
	Yes	53 (5.0)	17.10 (19.10)	0.24
Breastfeeding by 3-month	Mixed feeding (ref)	406 (38.4)	16.35 (17.35)	
	Only breastfed	527 (49.8)	13.90 (13.00)	<0.01
Doctors diagnosed colic 3-month	No (ref)	902 (85.3)	15.00 (14.80)	
	Yes	31 (2.9)	13.50 (20.1)	0.98
Infant abdominal pains 3-month	No (ref)	724 (68.5)	14.8 (14.65)	
	Yes	209 (19.8)	15.5 (16.05)	0.69

Supplementary Table S3. Mann–Whitney U-test stratified for infant biological sex of median infant saliva cortisol at three months age and maternal background factors and early life factors. Boy (n = 543) and Girl (n = 514).

		Boy			Girl		
		n (%)	median (IQR)	Sig.	*n* (%)	median (IQR)	sig.
Saliva cortisol (nmol/L)		543	15.5 (15.90)		514	15.10 (14.58)	0.69
Marital status	Married/co-habitant	500	15.70 (16.9)		479	15.2 (14.80)	
	Not living with a partner	43	11.70 (14.10)	0.13	35	12.10 (15.50)	0.48
Maternal education	University or higher	489	15.20 (15.50)		461	15.00 (14.70)	
	High school	54	19.70 (18.60)	0.28	53	15.60 (14.60)	1.00
Yearly family income	>600.000 NOK/SEK	459	15.40 (15.40)		435	15.00 (15.00)	
	Less than 600.000 NOK/SEK	84	16.15 (18.40)	0.49	79	15.20 (14.10)	1.00
Parity	First child	170	15.25 (19.00)		171	15.70 (15.60)	
	One or more children	373	15.50 (15.20)	0.95	343	15.00 (13.40)	0.85
Birth start	Spontaneous	420	15.20 (17.50)		383	15.90 (16.70)	
	Medical induced	119	16.20 (17.50)	0.66	121	13.80 (13.10)	0.16
Birth method	Vaginal	448	15.40 (15.20)		433	14.70 (13.50)	
	Caesarean section	95	15.50 (18.70)	0.92	81	17.40 (22.10)	0.09
PreventADALL intervention group	No intervention	104	14.45 (20.00)		110	16.10 (12.50)	
	Bath	96	12.45 (20.00)	0.48	116	14.55 (16.40)	0.35
	Food	125	18.00 (17.10)	0.16	102	15.70 (13.30)	0.88
	Bath and Food	110	17.60 (17.20)	0.13	99	15.90 (20.10)	0.94
Breastfeeding by 3-month	Mixed feeding	220	17.25 (17.10)		186	15.25 (17.70)	
	Only breastfed	263	13.20 (13.00)	<0.01	264	14.20 (13.10)	0.31
Doctors diagnosed colic 3-month	No	467	15.40 (14.50)		435	14.70 (15.20)	
	Yes	16	13.10 (18.60)	0.47	15	17.00 (22.60)	0.97
Infant abdominal pains 3-month	No	367	15.40 (15.30)		357	14.60 (13.70)	
	Yes	116	15.30 (12.50)	0.98	93	15.90 (18.90)	0.57
PSS Pregnancy 18 weeks	Low	429	15.50 (15.50)		417	15.60 (16,.70)	
	High	84	17.65 (19.00)	0.51	72	14.00 (10.50)	0.18
PSS Pregnancy 34 weeks	Low	449	15.50 (16.80)		434	14.95 (15.60)	
	High	71	17.90 (12.30)	0.42	62	15.45 (13.20)	0.66
PSS 3-month post-partum	Low	446	15.60 (15.50)		416	14.70 (14.50)	
	High	37	13.30 (15.50)	0.10	34	14.95 (20.90)	0.90

Supplementary Table S4. *T*-test of background characteristics and early life factors of included infants with saliva cortisol (*n* = 1057) and infants from the original PreventADALL mother/child cohort without saliva cortisol (*n* = 1337).

		Included (*n* = 1057)		Not included (*n* = 1337)		*p*-value
		n	mean (SD)	*n*	mean (SD)	
Maternal age, years		1057	32.8 (4.08)	1337	32.1 (4.18)	<.01
Gestational age, birth		1053	40.04 (1.35)	1337	40.06 (1.35)	0.81
Infant birth weight, kg		953	3.56 (0.54)	1301	3.53 (0.54)	0.23
PSS 18w		1003	20.91 (7.36)	1169	21.37 (7.16)	0.14
PSS 34 w		1016	19.97 (7.41)	1219	20.68 (7.19)	0.02
PSS 3 m		933	17.97 (7.27)	919	18.09 (7.21)	0.72
Saliva cortisol, maternal		531	34.19 (42.91)	191	30.20 (14.40)	0.21
Saliva cortisol, infant (inclusion criteria)		1057	32.1 (72.07)	na	na	na
		n	%	*n*	%	*p*-value
Maternal education	High school	107	(10.2)	134	(10.0)	
	University or higher	950	(89.8)	1203	(90)	0.93
Yearly income	Less than 600.000	164	(15.5)	176	(13.2)	
	>600.000	894	(84.5)	1162	(86.8)	0.12
Marital status	Married/cohabitant	979	(92.5)	1133	(84.7)	
	Not living with a partner	79	(7.5)	205	(15.3)	<.01
Parity	First child	341	(32.2)	317	(23.7)	
	One or more children	717	(67.8)	1021	(76.3)	<.01
Nicotine use by 3-month	no	880	(94.3)	869	(94.6)	
	yes	53	(5.7)	50	(5.4)	0.90
Induced labor	no	804	(77.0)	1039	(78.4)	
	yes	240	(23.0)	286	(21.6)	0.44
Birth method	Vaginal	881	(83.3)	1117	(83.5)	
	Caesarean section	176	(16.7)	221	(16.5)	0.97
Infant sex	Boy	554	(51.4)	713	(53.3)	
	Girl	514	(48.6)	625	(46.7)	0.39
Doctors diagnosed colic 3-month	no	902	(96.7)	891	(97.0)	
	yes	31	(3.3)	28	(3.0)	0.84
Infant abdominal pains by 3-m	no	724	(77.6)	713	(77.6)	
	yes	209	(22.4)	206	(22.4)	1.00
Breastfeeding by 3-month	Mixed feeding	406	(43.5)	435	(47.3)	
	Exclusive breastfed	527	(56.5)	484	(52.7)	0.11
Intervention group, bath	no	650	(75.3)	878	(76.0)	
	yes	213	(24.7)	277	(24.0)	0.76
Intervention group, bath and food group	no	441	(51.1)	615	(53.2)	
	yes	422	(48.9)	540	(46.8)	0.36

Supplementary Table S5. Spearman non-parametric correlations between maternal cortisol at 18 gestational weeks and maternal perceived stress in pregnancy at 18-, 34 weeks, and three-months infant age. Groups represent *all maternal saliva samples*, *morning sample* (05.00-10.59), *other sampling time* (11.00-04.59) and *missing sampling time.* Results are presented as numbers (*n*), correlation coefficient (r) and *p*-value.

	All maternal samples		*Morning sample*		*Other sampling time*		*Missing sampling time*	
	n	**r (*p*-value)**	*n*	**r (*p*-value)**	***n***	**r (*p*-value)**	***n***	**r (*p*-value)**
PSS 18 weeks	669	-0.08 (0.05)*	475	-0.08(0.08)	34	0.27 (0.18)	160	-005(0.56)
PSS 34 weeks	682	-0.04 (0.37)	494	-0.02 (0.60)	31	0.20 (0.28)	157	-0.12 (0.13)
PSS 3-month	586	-0.01 (0.77)	425	0.01 (0.79)	29	-0.07 (0.72)	132	0.04 (0.64)

*Not significant, 0.052
